# Finding the weakest link: mechanical sensitivity in a fish cranial linkage system

**DOI:** 10.1098/rsos.181003

**Published:** 2018-10-17

**Authors:** A. Baumgart, P. Anderson

**Affiliations:** 1 Department of Mechanical Science and Engineering, University of Illinois, Urbana, IL 61801, USA; 2 Department of Animal Biology, University of Illinois, Urbana, IL 61801, USA

**Keywords:** kinematics, linkages, modelling, suction feeding

## Abstract

Understanding the physical mechanics behind morphological systems can offer insights into their evolution. Recent work on linkage systems in fish and crustaceans has suggested that the evolution of such systems may depend on mechanical sensitivity, where geometrical changes to different parts of a biomechanical system have variable influence on mechanical outputs. While examined at the evolutionary level, no study has directly explored this idea at the level of the mechanism. We analyse the mechanical sensitivity of a fish cranial linkage to identify the influence of linkage geometry on the kinematic transmission (KT) of the suspensorium, hyoid and lower jaw. Specifically, we answer two questions about the sensitivity of this linkage system: (i) What changes in linkage geometry affect one KT while keeping the other KTs constant? (ii) Which geometry changes result in the largest and smallest changes to KT? Our results show that there are ways to alter the morphology that change each KT individually, and that there are multiple ways to alter a single link that have variable influence on KT. These results provide insight into the morphological evolution of the fish skull and highlight which structural features in the system may have more freedom to evolve than others.

## 1. Introduction

The laws of physics can exert a strong influence on the evolution of biological form [1–6]. Often, these physical laws are assumed to act as constraints, directing the flow of morphological evolution along certain paths [7]. However, the natural world is full of examples of functional convergence: morphologically distinct species that can perform equivalent mechanical functions. These patterns of convergence argue that the need for biomechanical systems to maintain functional fidelity (the ability to perform their mechanical function adequately) does not eliminate the ability of these systems to diversify. However, it is not clear whether the nature of biomechanical systems themselves can dictate how constrained or free they are to evolve morphologically. In this study, we explore the ability of a complex biomechanical system (the cranial linkage in *Salmo salar*) to be altered morphologically without compromising functional fidelity as determined by measures of kinematic transmission (KT).

In many mechanical systems, the relationship between structure and function often follows a pattern termed many-to-one mapping. Multiple geometries of a biomechanical structure may give the same mechanical result, enabling functionally neutral morphological evolution to occur. This pattern has been explored in the feeding system of teleost fishes [8–10]. Teleost jaw and opercular systems can be modelled as four-bar linkages, networks of four rigid bodies connected by joints in loops which transmit force and motion [11–14]. These linkage systems often have a nonlinear relationship between input and output rotations, and multiple forms can transfer the same amount of force or motion [10,11,15,16]. The many-to-one nature of this system may be a factor in the extensive morphological diversification seen in teleost fishes [17,18].

For many-to-one mapping to work, certain parts of the biomechanical system must be free to vary without altering overall function. At the same time, other parts of the system must be constrained by the need to maintain the system's function [4,5,19]. How alterations to different parts of a system vary in their influence on overall performance has been termed mechanical sensitivity [19]. Patterns of mechanical sensitivity indicate which components of a biomechanical system are free to evolve and which components are constrained by the need to maintain performance. Such patterns have been explored in both teleost jaws and mantis shrimp appendages at the evolutionary level [19,20]. Mechanical sensitivity has also been shown to potentially influence evolutionary rates in mantis shrimp [21]. While these evolutionary patterns are consistent with the hypothesis of mechanical sensitivity, little work has been done to actually identify the pattern of mechanical sensitivity of the components of the mechanism itself.

In this study, we explore the mechanical sensitivity of the cranial linkage system of Atlantic salmon (*Salmo salar*). Linkage mechanisms have been used to separately model movements of the oral jaws, hyoid and opercular mechanisms in fishes [11,12,22]. A recent study integrated these separate planar linkages into a more complex, three-dimensional linkage [23]. When linkage systems have movement occurring in three dimensions, simplifying the model to two dimensions can lead to inaccurate predictions of the original system's kinematics, especially when connected links may rotate in different planes [23].

To understand the mechanical sensitivity of this three-dimensional cranial linkage, we will test the effects of linkage geometry on various mechanical outputs (KT) using a previously published computer model of the linkage [23]. KT, the ratio of output rotation to input rotation in the linkage, is one of several functions of the cranial linkage; other functions include the transmission of force or torque, as well as the rate of expansion of the volume contained by the linkage. We will specifically address two questions: (i) What changes to the geometry of the linkage affect each individual mechanical output without affecting the others? This will allow us to find instances of many-to-one mapping for specific functional outputs. (ii) Which geometrical changes have the largest and smallest effects on each output? This will provide insight into which morphological features can be changed to optimize a specific output without compromising the other important outputs of the system. The results will also indicate which structural features of the fish cranial linkage have the most freedom to evolve, which can lead to new hypotheses regarding morphological diversity across species.

## 2. Methods

### 2.1. Computer model

To examine the effects of changing cranial linkage geometry on the prey capture ability of fish, we measured changes in multiple kinematic transmission outputs caused by geometry shifts in a three-dimensional linkage model. Generally, the biomechanical metric used to analyse biological four-bar linkages is kinematic transmission (KT), which has been shown to correlate well with observed feeding strategies of different fish species [15,24,25]. The mechanical performance of the three-dimensional linkage system examined here is composed of multiple KT values associated with the input rotation or translation of the neurocranium and hypohyal, respectively, and the output rotation of the oral jaws, hyoid and opercular. Using the published computer model of Olsen and Westneat [23,26], we simulate numerous, step-wise changes in cranial linkage geometries.

**Figure 1.**
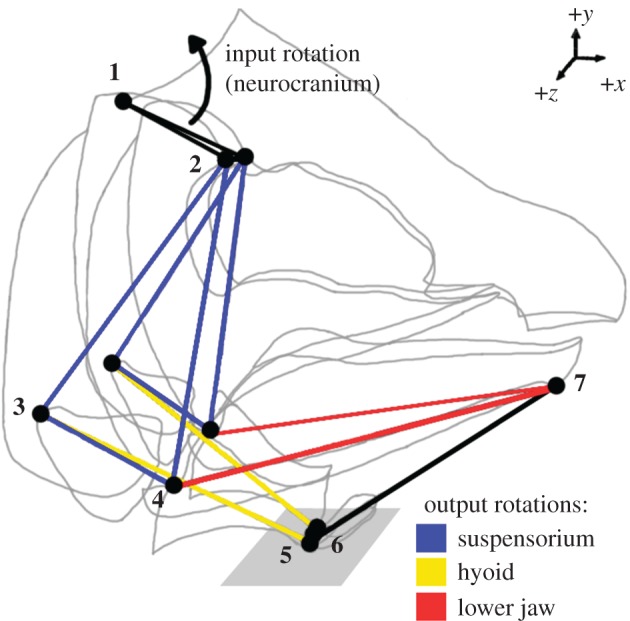
Published three-dimensional model from *linkR* [26]. Bars represent the links (coloured bars representing the three outputs) and grey lines outline the fish head. Numbered joints represent the neurocranium-vertebral column joint **(1)**, neurocranium-anterior suspensorium joints **(2)**, posterior ceratohyal-anterior suspensorium joints **(3)**, lower jaw-quadrate joints **(4)**, anterior ceratohyal-hypohyal joints **(5)**, midpoint of the hypohyal **(6)** and inferior aspect of the lower jaw symphysis **(7)**. The *x*–*y* plane is shaded around the hypohyal for reference.

The computer model used in this study is the *linkR* simplified fish cranial linkage model first published in Olsen & Westneat [23] (figure 1). The simplified model consists only of the joints of interest in the linkage system, connected by links as a stand-in for skeletal material. The joints included were the neurocranium-vertebral column joint, the left and right anterior neurocranium-suspensorium joints, the left and right posterior ceratohyal-suspensorium joints, the left and right lower jaw-quadrate joints, the left and right anterior ceratohyal-hypohyal joints, the midpoint of the hypohyal, and the inferior aspect of the lower jaw symphysis. The links represented the neurocranium, the left and right suspensorium, the left and right hyoid, the hypohyal, the left and right lower jaw, and the protractor hyoideus muscle, which is treated as a rigid link (as in the original model). Since this is a model of mouth opening, the protractor hyoideus is in tension and can be treated as rigid for simplification [23]. A set of joint coordinates are assigned to the model that correspond to the starting positions of each joint during a simulation (hereafter referred to as a *geometry*). The default geometry of these joint coordinates was based on landmark data from salmon specimens associated with the original model [23].

### 2.2. Measuring kinematic transmission for different geometries

**Figure 2.**
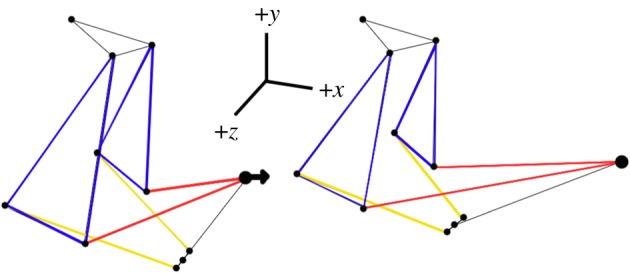
Link lengths were manipulated by moving each of the joints in the *x*, *y*, or *z* direction. This example changes the length of the lower jaw from 75% to 125% of the default length by shifting the lower jaw symphysis in the *x* (anterior-posterior) direction.

We performed a series of *modelling experiments* to examine the sensitivity of kinematic transmission to specific changes in link size and orientation during mouth opening. A single modelling experiment involved altering a single joint (or pair of symmetrical joints) and performing a series of simulations to understand how that change altered the performance of the linkage. During a *simulation*, an input rotation or translation is applied to the linkage and the joints move from their starting positions based on the degree of input rotation and linkage geometry. For each modelling experiment, a reference link is selected and the length of that link is increased or decreased by intervals of 1% to achieve a range from 75% to 125% of the original length taken from published specimens associated with the model [23]. Link lengths were calculated as the distance between joints, and therefore could not be altered independently of the joint coordinates. The starting positions of a single joint (or paired joints where necessary to maintain symmetry) connected to that reference link were then shifted in either the *x* (anterioposterior), *y* (dorsoventral) or *z* (lateral) direction to achieve the incremental changes in link length. Since the joints can be moved in many directions, each Cartesian direction represented in the model was tested separately to reduce the number of experiments. The result of this set-up was 51 geometries for each joint or set of joints in each Cartesian direction. As an example, the lower jaw symphysis joint was shifted in the *x* direction (anterioposteriorly) based on the percentage change to the length of the mandibular link (from 75% to 125% of the original mandible length) (figure 2). In a second modelling experiment, the lower jaw symphysis was shifted in the *y* direction (dorsoventrally) by the same percentage change in the mandibular link. For the joints which serve as endpoints for more than one link, the modelling experiments were conducted using geometries based on the percentage change to the length of each link at that joint. Note that for many of the modelling experiments, the 75–125% range was reduced based on geometric constraints of the linkage. These constraints resulted from linkage geometries that broke mathematically; the range was not reduced based on how realistic the model was biologically. Simulations at the endpoints of the range for each modelling experiment were tested individually to verify that none of the link lengths were forced to change length during mouth opening; for cases in which link lengths were non-constant, the simulation was removed from the modelling experiment, further reducing the testing range (see electronic supplementary material for a full list of ranges for each link geometry). There was no particular pattern to the joints affected by this range reduction.

**Figure 3.**
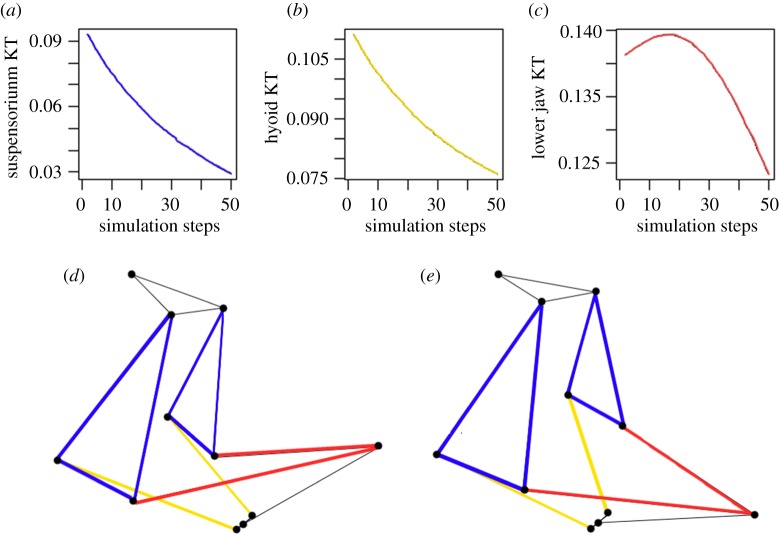
Suspensorium KT (*a*), hyoid KT (*b*) and lower jaw KT (*c*) measured over one simulation (50 steps of mouth opening, where 0 is completely closed (*d*) and 50 is completely open (*e*)). The average value from each plot was used to generate values for suspensorium, hyoid and lower jaw KT, respectively.

Kinematic transmission (KT), measured here as the ratio of output rotation to input rotation or translation, was measured for multiple input and output links. The inputs, tested separately, were either a 0.2 radian dorsal rotation of the neurocranium (actuated by epaxial musculature in fish) or a 3 mm purely posterior translation of the hypohyal (actuated by the hypaxial musculature in fish) [23]. These values were selected based on the default parameters of the original model. For the hypohyal input, the resulting KT calculated is not dimensionless, but has units of radians per mm. Note that in living fish it is likely that the epaxial and hypaxial musculatures work together during mouth opening [27]. However, in order to isolate effects, we analysed their inputs separately, as previously done [23]. The input magnitudes were held constant for all modelling experiments as the geometries were the focus of this study. Outputs tested were the rotations of the suspensorium, hyoid and lower jaw. Since KT varies throughout linkage rotation, it will change as the model moves, so for each simulation, the KT was measured at 50 evenly spaced points (intervals of 0.004 radians for neurocranium input or 0.06 mm for hypohyal input) (figure 3). As the lower jaw KT was the only one that showed distinct extrema (maxima or minima) during simulations, we used the average KT (KT: the average of the instantaneous KT across all 50 steps in a single simulation) as the standard metric for all the KTs measured (figure 3).

### 2.3. Defining mechanical sensitivity of kinematic transmission

**Figure 4.**
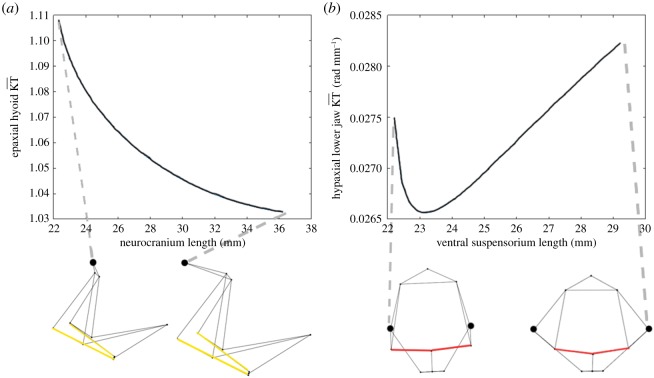
Two examples of curves generated by plotting the KT versus length of a reference link resulting from a specific joint shift. (*a*) Shows the hyoid KT versus the length of the neurocranium, which is altered by moving the neurocranium-vertebral column joint in the *x* (anterior-posterior) direction. (*b*) Shows the lower jaw KT versus the length of the ventral suspensorium, which is altered by moving the hyoid-suspensorium joints in the *z* (lateral) direction.

Rate of change of KT during a modelling experiment was approximated as the first derivative of KT with respect to the change in geometry over the course of the experiment. This was used as a metric for mechanical sensitivity, where a higher rate of change of KT associated with a given change in linkage geometry would indicate a higher mechanical sensitivity to said geometry change. From each simulation, KT was measured for each output (lower jaw, hyoid and suspensorium) (figure 3). A modelling experiment comprised all simulations run based on changing the geometry of a single point in one Cartesian direction. For instance, all simulations involving shifting the anterior neurocranium-suspensorium joints in the *x* direction count as a single modelling experiment (figure 4*a*), while changing the posterior ceratohyal-suspensorium joints in the lateral (*z*) direction counts as a separate experiment (figure 4*b*). For each modelling experiment, the lower jaw, hyoid and suspensorium KTs were measured over the range of geometries and plotted as a function of the link length they are based on, each point on the plot being the resulting KT from a single simulation (figure 4). The rate of change of the KT with respect to link length (ΔKT) was then approximated as a measure of how quickly the KT was changing as a result of shifting the geometry of that joint. The ΔKT values were compared across modelling experiments, with the modelling experiments having higher ΔKTs considered more mechanically sensitive than modelling experiments with lower ΔKTs.

Two forms of ΔKT were calculated for each modelling experiment. The instantaneous change in KT around the default geometry is calculated using equation (2.1), where *L* is the length of the link in question and *i* is indicative of the specific geometry used for the calculation:

2.1 $$\Delta KT_{L,i}=\frac{\bar{KT_{i+1}}-\bar{KT_{i-1}}}{L_{i+1}-L_{i-1}}.$$

**Figure 5.**
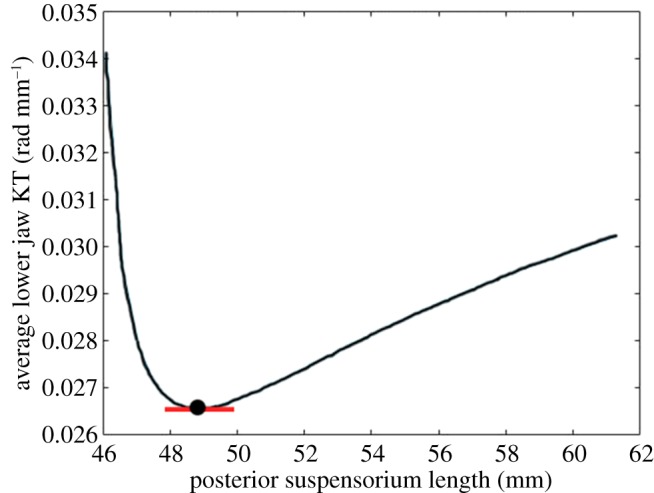
The hypaxial lower jaw KT versus the length of the posterior suspensorium, which is altered by moving the hyoid-suspensorium joints in the *z* direction. The point indicates the lower jaw KT at the default geometry. The red line indicates the instantaneous slope of the lower jaw KT at the default geometry.

This calculation may be made for any geometry in a modelling experiment; the default geometry is used here as it is based on specimen data. We also calculated an average ΔKT across the entire modelling experiment using the sum of the absolute values of the approximated derivatives at each geometry divided by the number of geometries. This is shown in equation (2.2):

2.2 $$\Delta KT_{L,\mathrm{average}}=\frac{\sum_{i}\Delta KT_{L,i}}{a},$$

where *a* is the number of geometries tested in the modelling experiment. This provided a measure of the average amount of change in KT per change in link length. The ΔKT values from both equations (2.1) and (2.2) were compared across modelling experiments to evaluate mechanical sensitivity. Two forms of ΔKT (instantaneous, from equation (2.1), and average, from equation (2.2)) were calculated because for some modelling experiments, the slopes of the KT versus link length plots varied substantially throughout the range of link lengths. For example, in the modelling experiment in which the length of the posterior suspensorium is altered by shifting the hyoid-suspensorium joints in the *z* direction, the slope of the lower jaw KT versus link length curve is zero at the default geometry, but non-zero for every other geometry tested (figure 5). As a result, the ΔKT*_L_*_,*i*_ and the ΔKT*_L_*_,average_ will lead to different interpretations of the mechanical sensitivity for this geometry change. Both types of ΔKT were examined for the identification of the most and least sensitive geometry changes as there was no clear indication of which metric was better for all modelling experiments.

### 2.4. Analysis

To determine how one KT output (jaw, hyoid or suspensorium) can be altered without affecting the others, we looked for geometry changes for which the ΔKT for two out of three of the outputs was equal to or close to 0. The largest ΔKT value in the dataset considered close to 0 was 2.04 × 10^−14^, which was separated by several orders of magnitude from the next largest value in the dataset, 8.66 × 10^−6^. The specific geometry change is then a way of changing only the KT which had a non-zero derivative. To determine what geometry changes had the least and greatest effects on each KT output (jaw, hyoid or suspensorium), the resulting ΔKTs for each output were examined separately. The ΔKTs were ranked from greatest to least, and the geometry changes corresponding to the highest ΔKTs (KT changing the fastest) and the lowest ΔKTs (KT changing the slowest) were noted.

## 3. Results

In each simulation, KT was measured over the range of motion occurring during initial mouth opening. Results for the default geometry are shown in figure 3. Both the hyoid and suspensorium KTs have no maxima or minima over the range tested, so the average KT (KT) is used for all three outputs as a standard metric for comparison between different linkage geometries.

For each set of modelling experiments, the KT was measured for each geometry and plotted against the length of a relevant link affected by that joint movement. A variety of trends of the dependence of each KT on a specific link's length were observed. The trends observed are generally nonlinear. Examples of these results can be seen in figure 4 and electronic supplementary material, figure S1.

The joint movement that had the largest effect on any of the KTs was shifting the lower jaw-quadrate joint in the *y* direction (dorsoventral). This joint movement affected the lower jaw KT but not the hyoid or suspensorium KT. For this modelling experiment, the lower jaw had a ΔKT*_L_*_,*i*_ of 0.4718 and a ΔKT*_L_*_,average_ of 0.7717, with the maximum and minimum lower jaw KTs being 4.247 and 1.409, respectively. The hyoid-suspensorium joints were most sensitive for the hyoid and suspensorium KT, with movement in the *z* direction (lateral) having the biggest effect on suspensorium KT and movement in the *y* direction (dorsoventral) having the biggest effect on hyoid KT.

### 3.1. Epaxial versus hypaxial input

In this study, simulations were done with either neurocranium input rotation (epaxial input) or hypohyal input translation (hypaxial input) to separate their effects on KT. Both lower jaw KT and suspensorium KT were more sensitive to changes in joint location with epaxial input than hypaxial input. For example, when the lower jaw-quadrate joints were shifted in the *y* direction, the modelling experiment with epaxial input resulted in the lower jaw ΔKT*_L_*_,*i*_ being 0.4718, while the modelling experiment with hypaxial input resulted in the lower jaw ΔKT*_L_*_,*i*_ being 0.0024 rad mm^−1^. That said, the basic patterns of mechanical sensitivity were the same for both inputs, so we will focus here on the epaxial input results (full hypaxial input results are in the electronic supplementary material).

### 3.2. Altering kinematic transmissions independently

The rates of change of $\bar{\mathrm{KT}}\;(\Delta \mathrm{KT})$ for all three outputs were examined for all modelling experiments. Geometries with zero or near zero values of ΔKT (orders of magnitude lower than the next highest rates of change) were noted. The effects of these geometry adjustments were compared between the three output links to identify the particular geometry adjustments that had minimal values of ΔKT for two of the three outputs while having a more significant value of ΔKT for the third output. Table 1 summarizes the results.

**Table 1.** Geometry changes that affected only one out of the three epaxial KTs measured.

	geometry changes	
KT isolated	joint adjustment	links affected
lower jaw	lower jaw-quadrate joints (*x*, *y*, *z*)	anterior and ventral suspensorium, lower jaw
	lower jaw symphysis (*x*, *y*, *z*)	lower jaw and protractor hyoideus
hyoid	hyoid-suspensorium joints (*y*) *still small effect on lower jaw and suspensorium KTs	posterior and ventral suspensorium, hyoid
suspensorium	neurocranium-suspensorium joints (*x*)	neurocranium, anterior and posterior suspensorium

Lower jaw KT was easily changed without affecting the hyoid or suspensorium KTs (denoted by high values of ΔKT for the lower jaw coupled with near zero values for the other two outputs). However, hyoid KT could not be completely isolated with a single geometry adjustment in this same way. It is possible that by combining geometry adjustments involving multiple joints with opposing effects on the lower jaw and suspensorium KTs, hyoid KT could be altered alone. Suspensorium KT was more easily isolated than the hyoid KT, although not to the extent of the lower jaw KT.

**Figure 6.**
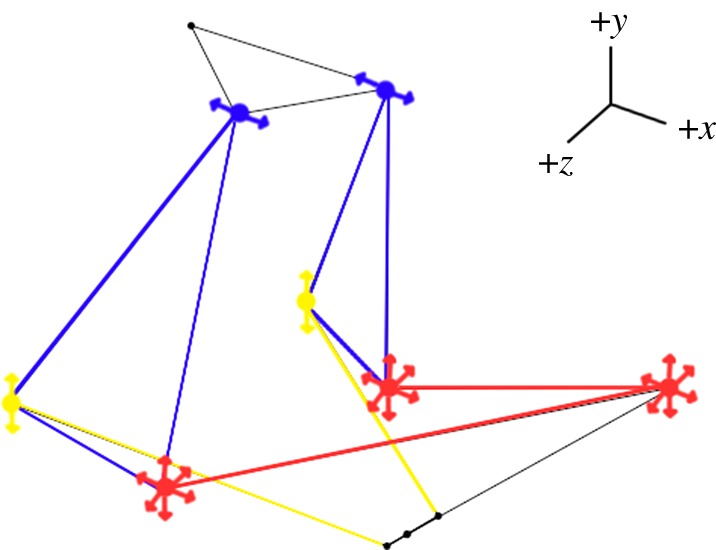
Arrows indicate which joint adjustments result in significant effects on one KT with minimal effect on the other two KTs. Red indicates lower jaw KT, yellow indicates hyoid KT, and blue indicates suspensorium KT. Note that for hyoid KT, the adjustment shown on the model had a non-zero effect on the other two KTs, while for the suspensorium KT and lower jawKT, adjustments could be made with zero effect on the other two KTs.

A visualization of these results is shown in figure 6.

### 3.3. Most and least sensitive ways to change each link length for each KT

For each link, each geometry adjustment affecting the length of that link was examined for each output separately. The highest and lowest magnitudes of ΔKT were noted for each output. These results are listed in table 2.

**Table 2.** Mechanical sensitivity of each link studied and its effects on epaxial KT. The highest and lowest ΔKT*_L_*_,*i*_ and ΔKT*_L_*_,average_ values for each reference link/output link combination demonstrate the ranges observed.

reference link	output link	geometry change	|ΔKT*_L_*_,*i*_|	ΔKT*_L_*_,average_
anterior suspensorium	lower jaw	l. jaw-quad. (*y*)	0.0823	0.1325
		l. jaw-quad. (*z*)	0.1916	0.0538
		neuro.-susp. (*y*)	8.38 × 10^−5^	9.20 × 10^−5^
		neuro.-susp. (*x*)	0.00	5.91 × 10^−15^
	hyoid	neuro.-susp. (*z*)	0.0238	0.0076
		neuro.-vert., neuro.-susp. (*y*)	0.0235	0.0235
		neuro.-susp. (*y*)	0.00260	0.0026
		neuro.-susp. (*x*)	9.85 × 10^−15^	1.02×10^−14^
		l. jaw-quad. (*x*, *y*, *z*)	0.00	0.00
	suspensorium	neuro.-susp. (*z*)	0.1012	0.039
		neuro.-vert., neuro.-susp. (*y*)	9.08 × 10^−4^	0.0016
		l. jaw-quad. (*x*, *y*, *z*)	0.00	0.00
posterior suspensorium	lower jaw	hyoid-susp. (*z*)	0.0692	0.0431
		neuro.-susp. (*y*)	0.00240	0.003
		neuro.-susp. (*x*)	2.04 × 10^−14^	1.15 × 10^−14^
	hyoid	hyoid-susp. (*y*)	0.0454	0.0435
		hyoid-susp. (*z*)	0.0613	0.0451
		neuro.-susp. (*y*)	0.00330	0.0034
		hyoid-susp. (*x*)	0.0041	0.0033
		neuro.-susp. (*x*)	1.02 × 10^−14^	1.40 × 10^−14^
	suspensorium	hyoid-susp. (*z*)	0.0957	0.0622
		neuro.-vert., neuro.-susp. (*x*)	0.00420	0.0049
ventral suspensorium	lower jaw	l. jaw-quad. (*y*)	0.162	0.253
		hyoid-susp. (*y*)	0.00650	0.0068
	hyoid	hyoid-susp. (*y*)	0.0710	0.069
		hyoid-susp. (*x*)	0.00270	0.0031
		l. jaw-quad. (*x*, *y*, *z*)	0.00	0.00
	suspensorium	hyoid-susp. (*z*)	0.107	0.0686
		hyoid-susp. (*x*)	0.0270	0.0284
		l. jaw-quad. (*x*, *y*, *z*)	0.00	0.00
hyoid	lower jaw	hypohyal (*y*)	0.1163	0.0786
		hyoid-susp. (*y*)	0.0071	0.0083
	hyoid	hyoid-susp. (*y*)	0.0773	0.067
		hyoid-susp. (*x*)	0.0031	0.0024
	suspensorium	hyoid-susp. (*z*)	0.0631	0.0863
		hypohyal (*y*)	0.0162	0.0117
lower jaw	lower jaw	l. jaw-quad. (*y*)	0.4718	0.7717
		l. jaw symph. (*y*)	0.0024	0.0034
	hyoid	l. jaw-quad. (*x*, *y*, *z*)	0.00	0.00
		l. jaw symph. (*x*, *y*, *z*)	0.00	0.00
	suspensorium	l. jaw-quad. (*x*, *y*, *z*)	0.00	0.00
		l. jaw symph. (*x*, *y*, *z*)	0.00	0.00

**Figure 7.**
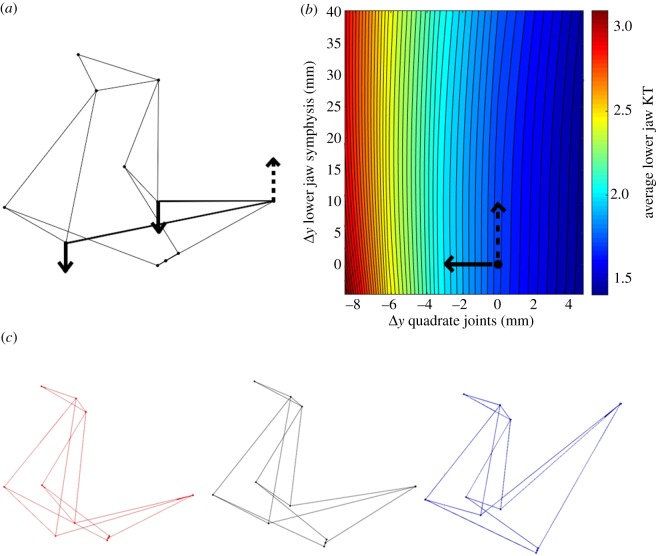
Results of changing the length of the lower jaw (bolded in (*a*)). The solid arrow indicates the most sensitive joint movement while the dashed arrow indicates the least sensitive joint movement (*a*). Maximum (*x*) and minimum (*y*) (non-zero) effects on lower jaw KT (*b*). The *x* and *y* axes represent two different ways of altering the geometry of a single link. The colour on the plot corresponds to the resulting KT associated with those shifts in geometry, with red being high and blue being low. The solid and dotted lines illustrate the change in KT values along the corresponding joint shifts in (*a*). The point shown on the contour plot indicates the default model from specimen data. (*c*) Shows (from left to right) the linkage corresponding to the bottom left corner of (*b*); the default linkage; the linkage corresponding to the top right corner of (*b*).

**Figure 8.**
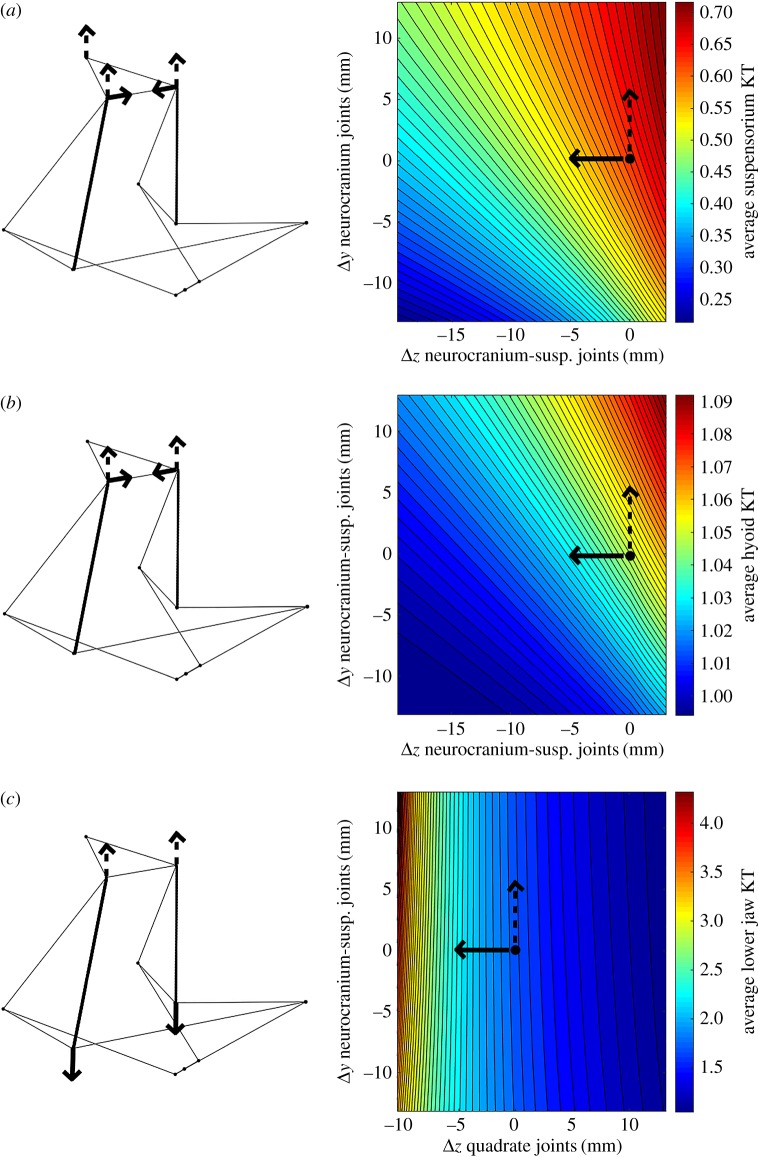
Results of changing the anterior suspensorium length (bolded in (*a*), (*b*), and (*c*)). Maximum (*x*) and minimum (*y*) (non-zero) effects on suspensorium KT (*a*), hyoid KT (*b*) and lower jaw KT (*c*). The *x* and *y* axes represent two different ways of altering the geometry of a single link. The colour on the plot corresponds to the resulting KT associated with those shifts in geometry, with red being high and blue being low. The solid arrows indicate the most sensitive joint movement while the dashed arrows indicate the least sensitive joint movement (directions of arrows on the models correspond to those on the plots). The points shown on the contour plots indicate the default model from specimen data.

The contour plots in figures 7 and 8 are examples of the differential effects of linkage geometry on ΔKT. For the lower jaw link, only lower jaw KT is affected, so only one gradient plot is needed. As shown, altering the jaw by moving the jaw symphysis in the *y* direction (dorsoventrally) has minimal effect on KT (denoted by the dotted line moving along the contours), while depressing the quadrate joint ventrally will have a much larger effect (the solid line cuts across the contours, showing rapid change in KT) (figure 7). Figure 8 shows similar results for how all three KTs are influenced by changes in the length of the anterior edge of the suspensorium. This visualization may be done for any combination of two geometry changes; we specifically show the comparisons of the maximum and minimum effect on KT as they represent the broad range of sensitivity of the system to changes in geometry.

## 4. Discussion

### 4.1. Mechanical sensitivity

Results from our modelling experiments show that individual geometry changes to the cranial linkage system of *Salmo salar* can have a range of influence on mechanical performance. Mechanical sensitivity was quantitatively described as a rate of change of KT per unit change in link length (ΔKT). Each modelling experiment was plotted as KT versus a specified reference link length relevant to the geometry change occurring in that experiment (figure 4). The slopes of these curves are the ΔKT, corresponding to the mechanical sensitivity of KT for that link geometry. Many of these plots are nonlinear, showing a change in mechanical sensitivity as the link length changes. Based on our quantitative definition, this suggests that mechanical sensitivity of a morphological component (e.g. link length) itself can change depending on the location of the shift in geometry.

For every link in the linkage system, we compared how KT changed relative to the specific geometries (joint shifts) that were used to generate change. The suspensorium links, hyoid links and lower jaw links all have multiple joints associated with them, and so there are many ways that the geometry of each of these links can be changed. There were some cases in which the same link length could be changed by moving different joints and result in opposite trends in KT (figure 8). These results indicate that KT is not sensitive to link length alone; the orientation of the links relative to each other and the positions of the joints themselves are also important. We did not test more than one joint movement at a time due to constraints on the time needed to run the simulations. It is likely that combinations of joint movements would lead to even more variation on patterns of KT. This suggests that when a biomechanical system is particularly complex, with multiple interconnected components, changes to individual components may be less significant to the system's output than shifts in geometry that alter multiple components simultaneously. In these experiments, we were sometimes unable to isolate changes in only one link at a time due to the interconnectedness of the linkage geometry itself. For example, there was no way to alter the length of the hyoid bar without also altering the length of at least one other link as well.

The interconnectedness of the linkage system may have implications for the integration of the head and jaws in teleost fishes. Integration, the interconnectedness of morphological structures, can arise from several sources including genetics, development, function and/or environment [28–30]. While a great deal of work has focused on the genetic and developmental underpinnings of integration, how function or biomechanics influences integration has been less understood [31]. Some studies have analysed morphological covariance among functionally relevant structures and speculated on how function may influence integration [32,33], but only a few have incorporated biomechanical data directly into their analyses [34,35].

The relationships highlighted by our modelling experiments may offer new insights into how the fundamental structure of a biomechanical system influences covariation between morphological components. Our experiments show that it is essentially impossible to alter the KT of the suspensorium without also altering hyoid KT and vice versa. The suspensorium and hyoid KTs could be viewed as a biomechanical module (a set of traits which are highly integrated with each other to the exclusion of other traits) that strongly influences the covariation between morphological components. This result is not surprising given that previous studies have modelled the suspensorium, hyoid and pectoral girdle as their own planar linkage system [11,19].

What is perhaps more surprising is the strong independence between the lower jaw and hyoid in our model, considering that the model assumes perfect coupling between these elements. The link between the jaw and hyoid represents the protractor hyoideus muscle and is modelled as never changing length during mouth opening (a reasonable simplification as the link will always be in tension during this action). We lack the kinematic data to currently test whether this assumption of perfect coupling is justified. However, it is noteworthy that if the two links were not perfectly coupled, it would simply increase the independence of the lower jaw relative to the rest of the system. While coupling of the jaw and hyoid during suction feeding has not been examined closely in salmonids, there is evidence for the independence of these two elements after prey capture. A lack of correlation between jaw and hyoid displacement during open-mouth chewing behaviours has been shown in salmonids [36]. There is a reported two-dimensional opercular linkage between the lower jaw and suspensorium in some teleosts [11,37]. However, this linkage appears to have three degrees of freedom, allowing greater motion [38].

Since the lower jaw KT can be altered independently from the suspensorium and hyoid KTs, we might expect that the morphological covariance of the lower jaw with the suspensorium and hyoid would be weaker than the morphological covariance of the suspensorium and hyoid with each other. Comparing trends in KT changes with trends in morphological changes among the three structures may offer deeper insight into how the nature of a biomechanical system can influence morphological covariance.

How the functional integration outlined above will interact with other measures of integration (genetic or developmental) is unclear. The few studies that have attempted to synthesize functional integration with developmental integration have done so by assessing their respective influences on empirical patterns of morphological covariation [32,39–43]. Few studies have tried to link genetics or developmental processes with specific functional consequences [35]. Our results show that in linked systems such as the salmonoid cranial linkage, it is difficult to alter one functional output without altering others as well, leading to potential constraints on morphological evolution even when genetic or developmental data may point towards a lack of integration. On the other hand, the lack of strong integration between the jaw and hyoid outputs may not matter if the jaw system is integrated at the genetic or developmental levels. The relative influence of integration at these different levels on evolutionary processes has not been explored empirically, but our results here offer a new method for comparing functional integration with integration at other levels.

### 4.2. Evolutionary implications

Our modelling results give further insights into the many-to-one mapping of the teleost cranial linkage system. During our experiments, there were many ways we could change the linkage geometry that would alter one KT individually while keeping the other two KTs constant. This was most apparent in the case of the lower jaw KT, but all three KTs had some way of achieving this (figure 6; table 1). As an example, changes involving the lower jaw link affected the lower jaw KT but not the hyoid or suspensorium KTs. This implies that the lower jaw can evolve morphologically without having an impact on the KT of the hyoid and suspensorium. The ability to separate the lower jaw from the suspensorium and hyoid may be advantageous as both the suspensorium and the hyoid play important roles in suction feeding [44], while the lower jaw has other functions it is involved in, such as reach, gape and bite force [15]. This is not to suggest that the hyoid and suspensorium are not multi-functional as well; the lower jaw may simply have stronger selective pressures outside of suction feeding. The separation of the lower jaw from the other parts may allow fish to modify performance in the other lower jaw functions without sacrificing the feeding mechanics of the suspensorium and hyoid. This has been observed in the comparison of biting and suction feeding performance in clariid catfishes [45].

On the other hand, our experiments also showed that a single joint shift could often affect more than one KT (note in figure 8 that moving the neurocranial-suspensorium joints medially exerted strong influence on both the suspensorium and hyoid KTs). In these cases, it may be possible to ‘improve’ more than one function simultaneously using a single morphological change. In some experiments, a geometry change would result in multiple KTs increasing, while others would result in an increase in one KT and a decrease in another. This suggests that there does not necessarily have to be a trade-off in the evolution of multiple functions, in which optimizing one KT results in a different KT becoming suboptimal. It is important to note that KT is not the only relevant output of a linkage system, particularly in linkage systems associated with fish feeding. Morphological changes may not directly influence these KT measures but may affect other important outputs such as jaw protrusion or aspects of suction feeding such as flow speed control, strike efficiency and mouth displacement [46,47]. The ability for a single change in morphology to alter multiple performance metrics could also result in instances of exaptation; an organism is able to take on a new ecological role by co-opting structures it has already evolved [48]. However, the results of this study need to be compared with data for a range of fish clades before hypotheses of exaptations can be made with any confidence. Work to that end would be instructive, as it could reveal if the cranial linkage has evolved to improve multiple functions of the linkage together or if it has instead followed a pattern of functional trade-offs.

Overall, the results of our modelling experiments suggest that complex biomechanical systems, such as the fish cranial linkage, may actually ease constraints on morphological evolution due to the requirement of functional fidelity. When looking at the values of KT calculated by the model, there were no specific geometry changes required to achieve a specific KT value. This absence of a unique solution to produce a desired output means that if a certain KT output value were heavily advantageous, the fish is not restricted from evolving morphologically. This is partly due to the nonlinear nature of the relationship between geometry shifts and KT shifts seen in our simulations. There is not one, single pattern in the relationship between KT and reference link length, but numerous patterns (figure 3). Some curves are roughly linear, while others appear to have inverse, quadratic, or polynomial trends. There were also some simulations in which there was a local minimum or maximum KT value. This pattern of nonlinearity was seen for all combinations of input and output as well as for all links altered. There are numerous ways for evolution to tinker with this linkage system and attain any number of results for the various performance metrics.

## 5. Conclusion and future work

Examining the physics behind biological systems allows for a deeper understanding of how the need to maintain functional fidelity may constrain morphological evolution. The cranial linkage system of teleost fishes is a multi-part mechanism that plays an important role in feeding. By measuring the effects of varying linkage geometry on the mechanical outputs of the linkage system in *Salmo salar*, we have identified morphological changes that influence a single performance output in isolation from others, as well as which morphological changes have the greatest and least influence on each performance output. Numerous geometrical changes can result in equivalent kinematic transmission values, a clear example of many-to-one mapping. However, not all structural changes were equivalent in terms of the level of influence on mechanical outputs measured, an example of differential mechanical sensitivity. Furthermore, altering the lower jaw KT was easily done without altering the hyoid or suspensorium KTs, while the latter two KTs are much harder to isolate. This suggests biomechanical integration between the hyoid and suspensorium, with the lower jaw having fewer evolutionary constraints. Recognizing patterns such as this may offer a new way to incorporate functional or biomechanical data into studies of integration and modularity at multiple levels.

All of our results are based on manipulations of a theoretical model, but they provide a useful tool for future work on the evolution of cranial morphology in the salmonoid clade. In a sense, these modelling results provide a road-map for examining which aspects of morphology should diversify and which should be constrained across the group. Previous work on linkages in both fish and mantis shrimp have shown that evolutionary rates can be influenced by patterns of mechanical sensitivity [21,49]. Future work on salmonoids could use the data from this paper to make hypotheses of what parts of the system should show higher rates of evolution and larger disparity compared with others. If these predictions match, it would be a strong indication of how biomechanical integration can influence evolutionary processes.

While this study is focused solely on the salmonoid linkage system, our modelling approach is applicable to any multi-part system provided its function is well constrained. For instance, this model could be modified in the future to accommodate the structure of other fish cranial systems to see if the same patterns of mechanical sensitivity emerge. Linkage systems have also been identified in bird crania [23]. Since birds do not use their cranial linkages for suction feeding, we might expect different patterns of mechanical sensitivity than those seen in fishes. Our modelling approach could be used to identify similar or divergent patterns of mechanical sensitivity in disparate clades.

Mechanical sensitivity as tested here is not restricted to linkages alone. Mantis shrimp use a linkage system in conjunction with a power-amplified spring system to attain high-speed strikes [50–52]. The potential mechanical sensitivity of the linkage system in this clade has been explored at the evolutionary level [50–52]. However, the modelling techniques used here could be modified to include the linkage along with the spring system to test for sensitivity across various outputs (speed, energy, force). Any multi-part biomechanical system such as this could be analysed, allowing for major comparative analyses across classes to identify potentially common patterns in mechanical sensitivity and integration.

This study represents a theoretical model of the cranial linkage system of a single fish taxon (*Salmo salar*) focusing on just one type of output, kinematic transmission. There is much more work to be done to examine mechanical sensitivity patterns in other multi-part biomechanics systems, be they fish clades or non-teleost systems such as the linkage and spring systems in mantis shrimp. Our modelling exercise here represents a first attempt to test aspects of mechanical sensitivity in a well-defined system and the results show the promise of this approach for future studies.

## Supplementary Material

Baumgart and Anderson SI

## Supplementary Material

Input data for the cranial linkage model

## Supplementary Material

Results file

## Data accessibility

The model used in this study is part of the *linkR* package for R, available at: https://cran.r-project.org/web/packages/linkR/index.html. The input coordinates used for all of the modelling experiments can be found in the electronic supplementary material.
