# Supplementary material for: Finding the weakest link: mechanical sensitivity in a fish cranial linkage system
Source: R Soc Open Sci. 2018 Oct 17;5(10):181003. doi: 10.1098/rsos.181003 (PMC6227944; doi:10.1098/rsos.181003)
Supplement: Baumgart and Anderson SI [file rsos181003supp1.docx]

Supplementary Information for:

**Finding the weakest link: Mechanical sensitivity in a fish cranial linkage system*.***

Baumgart^1^, A. and Anderson^2^, P.

1. Department of Mechanical Science and Engineering, University of Illinois, Urbana, IL 61801

2. Department of Animal Biology, University of Illinois, Urbana, IL 61801

*Corresponding author: andersps@illinois.edu

Contents:

Notes for file *SI_Inputs.xlsx*---------------------------------------------------------------Pg. 2

Notes for file SI_Results.xlsx--------------------------------------------------------------Pg. 3

Figure S1---------------------------------------------------------------------------------------Pg. 4

Notes for the Supplementary file: S1_*Inputs.xlsx*

This file contains the coordinate inputs for the *linkr* model used for modeling experiments.

**“default input and lengths” sheet:**

This sheet shows the default coordinates (x,y,z) for every joint. Also shown are the default link lengths for each link in the model.

**other sheets:**

Each sheet shows the modified coordinates for a single joint modified in one cardinal direction over the course of a single modelling experiment.

For each sheet, the name is the joint and direction. If a joint shift would affect more than one possible reference link, the specific reference link being used for the sheet is indicated with a capital letter (N = neurocranium, Sa = suspensorium (anterior), Sp = suspensorium (posterior), Sv = suspensorium (ventral), H = hyoid, Hp = hypohyal, J = lower jaw)

For example, the first sheet is labeled **nc_vc_x**. This sheet shows the input coordinates for the neurocranium-vertebral column joint as it is shifted in the x-axis by steps equivalent to 1% change in the reference link (Neurocranium).

The second sheet is labeled **nc_su_a_L_nc_su_a_R_nc_vc_x_Sa**. The sheet shows modification in the x-axis of three joints simultaneously: the neurocranium-vertebral column joint and the left and right neurocranium-anterior suspensorium joints. The reference link used is the suspensorium.

On each sheet, the first column indicates the joint being modified. The second through fourth columns are the x, y, and z coordinates for the joint in question. Each row represents the coordinates for that joint in a single simulation. As you move down the rows, the joint is shifted in the given direction by an amount that causes a 1% change in length of the reference link.

Shaded rows indicate coordinates not used due to geometric/mathematical constraints on the linkage (i.e., achieving that link length by moving that joint would require imaginary joint displacements; the would-be “imaginary” coordinates are replaced with 0’s so that the spreadsheet can be read into the simulation without raising errors, although those rows are not used in the modelling experiments).

**Joint Abbreviations:**

nc_vc: neurocranium-vertebral column joint

nc_su_a_L/nc_su_a_R: neurocranium-anterior suspensorium joints (left/right)

pc_su_L/pc_su_R: posterior ceratohyal-suspensorium joints (left/right)

lj_qd_L/lj_qd_R: lower jaw-quadrate joints (left/right)

ac_hy_L/ac_hy_R: anterior ceratohyal-hypohyal joints (left/right)

hy_mid: midpoint of hypohyal

lj_sy_inf: inferior aspect of lower jaw symphysis

Notes for the Supplementary file: S2_*Results.xlsx*

This file contains the *ΔKT* results for every modelling experiment performed. Each row represents the results for one *KT* measure during a single modelling experiment. For example, the first row shows the *ΔKT* results for lower jaw *KT* when the anterior suspensorium link is modified by moving the lower jaw quadrate joint in the x direction.

*shaded rows indicate 0 or ~0 *ΔKT*

*directions correspond to manuscript/figures

**Joint Abbreviations:**

nc_vc: neurocranium-vertebral column joint

nc_su_a_L/nc_su_a_R: neurocranium-anterior suspensorium joints (left/right)

pc_su_L/pc_su_R: posterior ceratohyal-suspensorium joints (left/right)

lj_qd_L/lj_qd_R: lower jaw-quadrate joints (left/right)

ac_hy_L/ac_hy_R: anterior ceratohyal-hypohyal joints (left/right)

hy_mid: midpoint of hypohyal

lj_sy_inf: inferior aspect of lower jaw symphysis


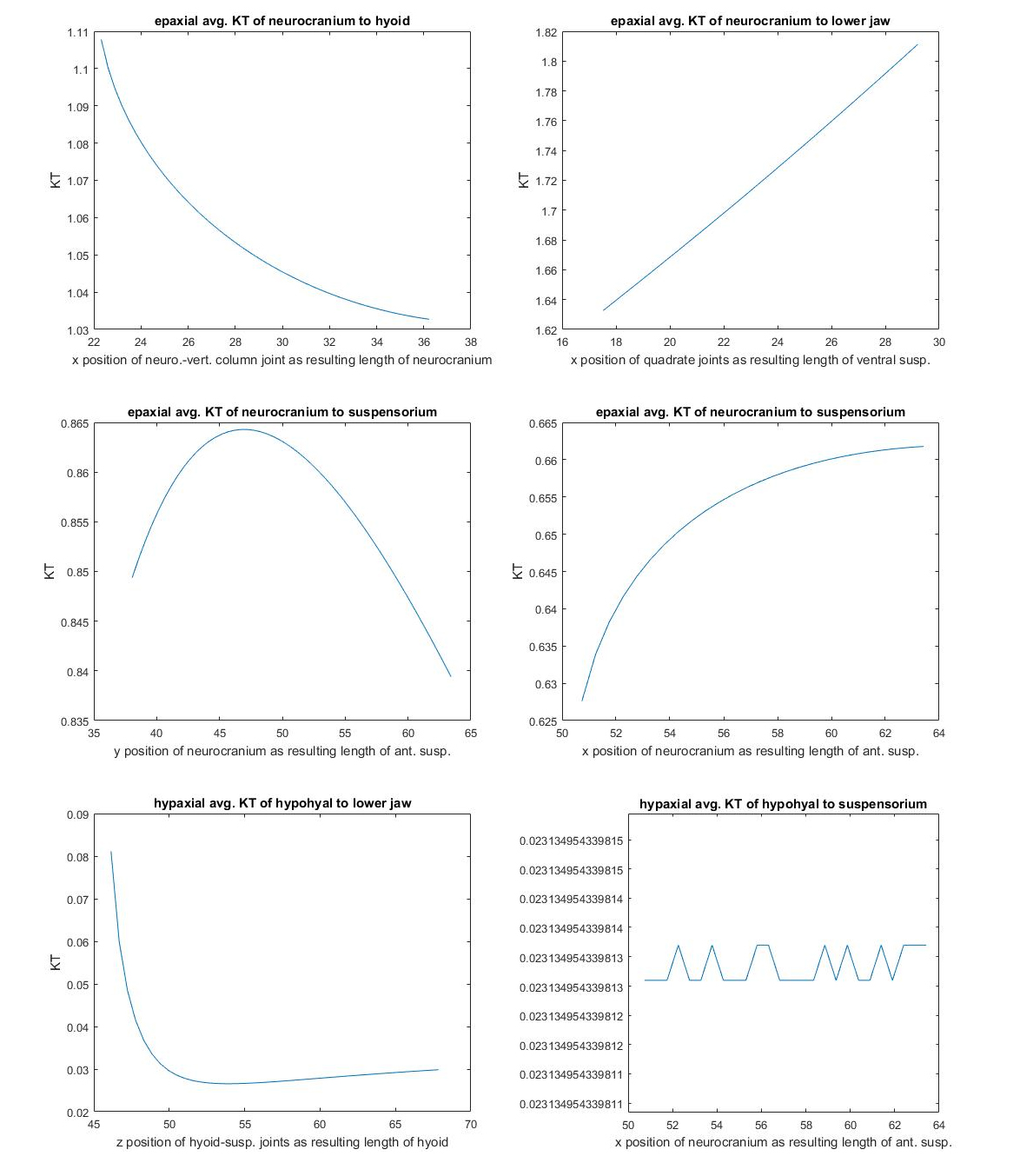


Figure S1: $\bar{KT}$ curves representing the variety of trends observed across all modelling experiments. All curves generated during the modelling experiments conformed to one of these basic shapes. For each plot, the x-axis represents the length of the reference link, which is altered by shifting a joint or set of joints in the specified direction. The y-axis shows the $\bar{KT}$ for each geometry tested throughout the modelling experiment.
